# Enhanced Adsorption of Methyl Orange from Aqueous Phase Using Chitosan–Palmer Amaranth Biochar Composite Microspheres

**DOI:** 10.3390/molecules29081836

**Published:** 2024-04-18

**Authors:** Guiling Chen, Yitong Yin, Xianting Zhang, Andong Qian, Xiaoyang Pan, Fei Liu, Rui Li

**Affiliations:** School of Biological Science, Jining Medical University, No. 669 Xueyuan Road, Donggang District, Rizhao 276826, China; chenguiling@mail.jnmc.edu.cn (G.C.); yytsdml@163.com (Y.Y.); 13589619982@163.com (X.Z.); 13310637723@163.com (A.Q.); pan2071078420@163.com (X.P.)

**Keywords:** biochar, chitosan, invasive weed, dye treatment, methyl orange

## Abstract

To develop valuable applications for the invasive weed Palmer amaranth, we utilized it as a novel biochar source and explored its potential for methyl orange adsorption through the synthesis of chitosan-encapsulated Palmer amaranth biochar composite microspheres. Firstly, the prepared microspheres were characterized by scanning electron microscopy and Fourier transform infrared spectroscopy and were demonstrated to have a surface area of 19.6 m^2^/g, a total pore volume of 0.0664 cm^3^/g and an average pore diameter of 10.6 nm. Then, the influences of pH, dosage and salt type and concentration on the adsorption efficiency were systematically investigated alongside the adsorption kinetics, isotherms, and thermodynamics. The results reveal that the highest adsorption capacity of methyl orange was obtained at pH 4.0. The adsorption process was well fitted by a pseudo-second-order kinetic model and the Langmuir isotherm model, and was spontaneous and endothermic. Through the Langmuir model, the maximal adsorption capacities of methyl orange were calculated as 495.0, 537.1 and 554.3 mg/g at 25.0, 35.0 and 45.0 °C, respectively. Subsequently, the adsorption mechanisms were elucidated by Fourier transform infrared spectroscopy and X-ray photoelectron spectroscopy investigations. It is indicated that electrostatic interactions, hydrogen bonding, π–π interactions and hydrophobic interactions between methyl orange and the composite microspheres were pivotal for the adsorption process. Finally, the regeneration studies demonstrated that after five adsorption–desorption cycles, the microspheres still maintained 93.6% of their initial adsorption capacity for methyl orange. This work not only presents a promising method for mitigating methyl orange pollution but also offers a sustainable approach to managing Palmer amaranth invasion.

## 1. Introduction

Methyl orange (MO), identified as 4-[[(4-dimethylamino)phenyl]-azo]benzenesulfonic acid sodium salt, is a member of the azo dye family [1]. It is widely used in diverse industries, including textiles, leather, papermaking, cosmetics and food processing [2]. Long-term exposure to this dye can result in vomiting, diarrhea, cancers and even death [3,4]. Thus, the unregulated discharge of MO can cause significant threats to human health and also environmental safety. However, MO has good stability, high solubility and low biodegradability in aqueous solutions [5], so its removal from water bodies is a challenging endeavor.

Adsorption separation is a typical method for dye removal, due to its simple design and process and low operational costs [6]. Furthermore, this method facilitates the potential reuse of dyes [7]. The development of new adsorbents has always been a prominent issue in the field of adsorption separation [8,9,10]. Currently, a large proportion of the literature has reported the adsorption removal of MO from aqueous solutions [11,12,13]. Among the various adsorbents employed, biochar, which is a carbonaceous substance derived from the pyrolysis of biomass in oxygen-deprived or oxygen-free environments, has emerged as a subject of increasing interest [14]. This interest stems from its large specific surface area, multiple functional groups and good environmental friendliness [15]. At present, biochar sourced from chicken manure [16], rice husk [17], sheep manure [18], mandarin peel [19], pine cone [20], date palm petioles [21], sawdust, bamboo and palm [22] have been investigated for MO adsorption. However, the majority of these biochar adsorbents exhibit limited MO adsorption capacities (lower than 100 mg/g) [16,17,18,19,20,22]. Hence, there exists a necessity to develop biochar-derived adsorbents with enhanced MO adsorption capabilities to ensure effective dye removal.

The origin of biochar significantly influences its adsorption capacity [22,23,24]. Palmer amaranth (*Amaranthus palmeri* S. Watson) is an invasive agricultural weed widespread across more than 45 countries [25]. It poses a significant threat to agricultural ecosystems due to its capacity for aggressive competition with crop plants for essential resources [26]. To mitigate the deleterious impact of Palmer amaranth on agriculture and explore its potential reuse, we propose its utilization as a novel biochar source for MO adsorption. Various techniques, such as slow pyrolysis, microwave-assisted pyrolysis, fast pyrolysis, gasification and flash carbonization, have been employed in biochar preparation [27,28]. Among these, slow pyrolysis stands out as the preferred method, as biochar produced through this process typically exhibits heightened porosity and increased surface functional groups, resulting in superior adsorption properties [29,30]. In this study, slow pyrolysis will be employed for Palmer amaranth biochar (PABC) production.

Furthermore, the modification of biochar is also very useful in increasing its adsorption capacity [31]. For instance, Park et al. [19] and Cheng et al. [32] employed modifications with NH_4_Cl, ZnCl_2_ and Fe^2+^ to enhance the adsorption capacity of MO. Compared to chemical modifications, chitosan-based modification presents a more environmentally sustainable option [33]. Thus, the synthesis of chitosan—PABC composite microspheres will be pursued by encapsulating biochar within chitosan to improve the adsorption capacity of MO and enhance the reusability of biochar [34]. This absorbent will facilitate research across four key domains: (1) the characterization of the composite microspheres using scanning electron microscopy (SEM), Fourier transform infrared spectroscopy (FTIR) and the Brunauer–Emmett–Teller (BET) method; (2) the assessment of the adsorption efficiency for MO removal under diverse solution conditions; (3) the elucidation of the adsorption mechanism via kinetic and thermodynamic analyses, as well as FTIR and X-ray photoelectron spectroscopy (XPS); and (4) the evaluation of MO desorption from the composite microspheres and their subsequent reusability. These endeavors collectively aim to furnish an innovative absorbent with a superior adsorption capacity for MO removal and Palmer amaranth recycling.

## 2. Results and Discussion

### 2.1. Characterization Results

**Figure 1:**
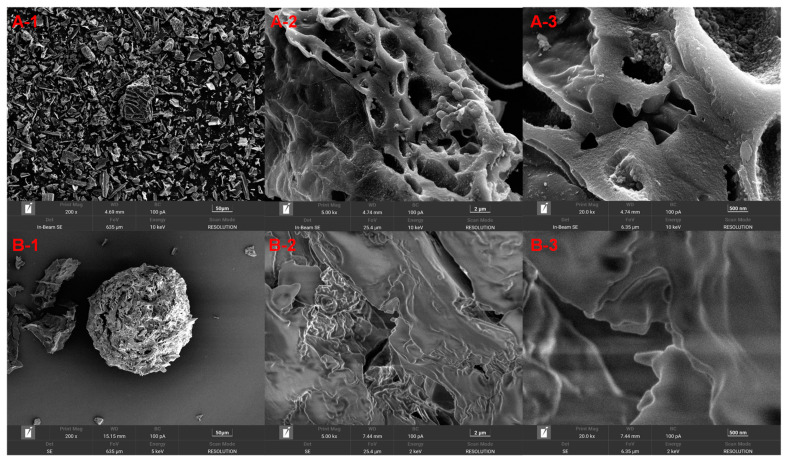
SEM images of PABC ((**A-1**), amplification ×200; (**A-2**), amplification ×5000; (**A-3**), amplification ×20,000) and chitosan–PABC composite microspheres ((**B-1**), amplification ×200; (**B-2**), amplification ×5000; (**B-3**), amplification ×20,000).

In this study, SEM was utilized to elucidate the microstructural attributes of PABC and chitosan–PABC composite microspheres. The SEM images, presented in Figure 1, reveal the microstructural variations at different magnifications. The PABC samples demonstrate a flat, stratified morphology with discernible folds and crevices upon enhanced magnification, indicative of a material that is both flexible and porous (Figure 1A). Conversely, the chitosan–PABC composite microspheres are characterized by a more heterogeneous, rugged, and porous surface topology (Figure 1B), suggesting an increased surface area, which is advantageous for adsorption applications. Supplementary to the morphological analysis, the BET surface area analysis further quantifies the surface characteristics of the composite microspheres, revealing a surface area of 19.6 m^2^/g, a total pore volume of 0.0664 cm^3^/g and an average pore diameter of 10.6 nm (refer to Figure S1). These findings not only corroborate the morphological observations from Figure 1 but also confirm the presence of mesopores within the microspheres.

**Figure 2:**
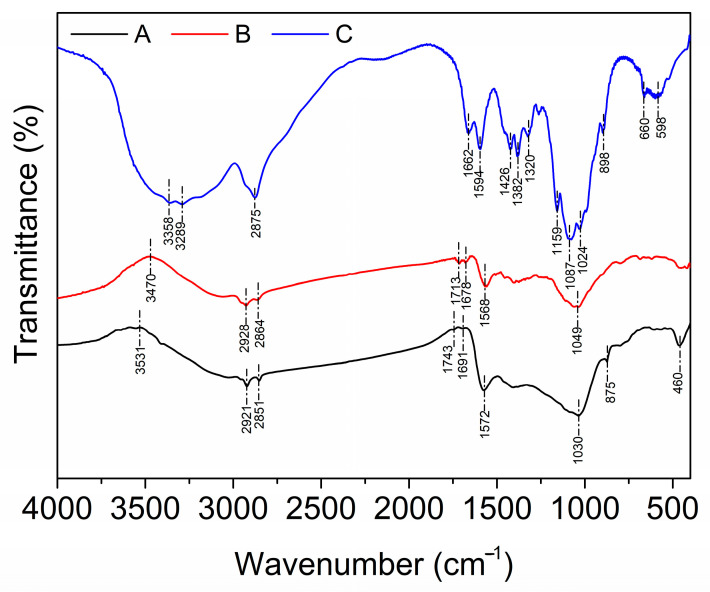
FTIR spectra of PABC (A), chitosan–PABC composite microspheres (B) and chitosan (C).

Functional group characterization on the surfaces of the chitosan–PABC composite microspheres was conducted through FTIR spectroscopy with chitosan and PABC as references. The outcomes of this analysis are delineated in Figure 2. From the FTIR spectrum for PABC, the upwards convex peak at 3531 cm^−1^ could be attributed to the stretching of O-H, typically from hydroxyl groups and hydrogen bonds. Furthermore, the stretching vibrations of C-H from aliphatic chains (2921 and 2851 cm^−1^), C=O from carboxyl groups (1743 cm^−1^), C=O from ketone or aldehyde groups (1691 cm^−1^), C=C (1572 cm^−1^) and C-H (875 cm^−1^) from aromatic rings, C-O from hydroxyl or ether bonds (1030 cm^−1^) and metal–oxygen bonds from residual minerals (460 cm^−1^) were also observed [35,36,37]. These results indicate the carbonaceous structure of biochar. Chitosan’s FTIR spectrum was quite distinct. It was characterized by the stretching vibrations of O-H (3358 cm^−1^), N-H from amine groups (3289 cm^−1^), C-H (2875, 1426 and 1382 cm^−1^), C=O from the amide I band (1662 cm^−1^), N-H from the amide II band (1594 cm^−1^), C-N (1320 cm^−1^), C-O (1024 cm^−1^), C-O-C (1159 and 1087 cm^−1^) and beta-glucoside bonds (898 cm^−1^) [38]. In addition, the peaks at 660 and 598 cm^−1^ were typically associated with out-of-plane deformations or wagging vibrations of the sugar rings.

Upon comparison of the FTIR spectra between the composite microspheres and PABC, a noticeable shift was observed in the broad, upwards convex peak to 3470 cm^−1^, indicative of hydrogen bonding interactions between hydroxy groups from the biochar and amino groups from chitosan. The observed peaks corresponding to the stretching vibrations of the C=O groups (1713 and 1678 cm^−1^) in the composite microspheres’ spectrum exhibited an increase in area and underwent blue shifts relative to those in the PABC’s spectrum. This is a clear indication of crosslinking between the biochar and chitosan in the composite beads. Furthermore, characteristic peaks associated with biochar, such as those for the stretching vibrations of C-H (2921 and 2851 cm^−1^), C=C (1568 cm^−1^) and C-O (1030 cm^−1^), were discernible in the spectrum of the composite microspheres, albeit with slight shifts. In addition, the peaks representing C-H (875 cm^−1^) and the metal–oxygen bonds (460 cm^−1^) within the biochar spectrum were markedly diminished in the composite microspheres’ spectrum. These alterations suggest a level of interaction between the functional groups of biochar and the saccharide structure of chitosan [39,40].

### 2.2. Influent Factors

#### 2.2.1. Effect of Initial pH

**Figure 3:**
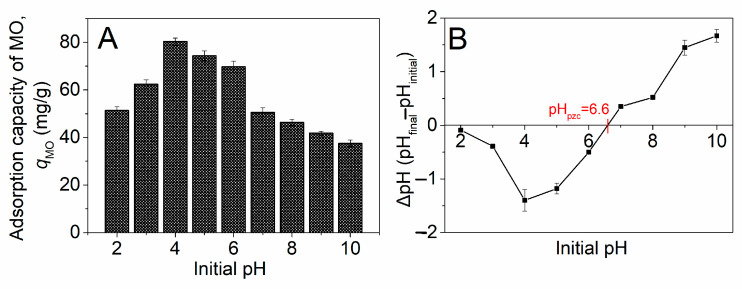
Effect of initial pH on adsorption capacity of MO on chitosan–PABC composite microspheres (**A**) and isoelectric point of chitosan–PABC composite microspheres obtained by pH drift method (**B**).

pH often has a significant effect on the adsorption performance of chitosan–biochar composite microspheres [34]. In this context, the initial pH’s impact on the adsorption capacity of MO of the chitosan–PABC composite microspheres was investigated under the conditions of a microsphere dosage of 100 mg, an initial MO concentration of 50 mg/L and temperature of 25.0 °C without the addition of any salts. According to the findings depicted in Figure 3A, the adsorption capacity (*q*_MO_) escalated from 51.3 ± 1.6 mg/g to 80.3 ± 1.5 mg/g as the pH was augmented from 2 to 4, subsequently diminishing to 37.5 ± 1.4 mg/g with the pH further increasing. Consequently, pH 4.0 was identified as the optimum for the adsorption of MO on the chitosan–PABC composite microspheres.

With a pKa value of 3.7 in aqueous solutions [41], the MO molecules began to dissociate into negatively charged ionic states upon the pH surpassing 3.7. Figure 3B elucidates that the isoelectric point of the chitosan–PABC composite microspheres was at pH 6.6, rendering the microspheres positively charged at pH values below 6.6. Accordingly, the electrostatic attraction between the positively charged microspheres and the negatively charged MO molecules intensified the adsorption in the pH range of 3.7 to 6.6, as evidenced by the relatively high *q*_MO_ values at pH levels of 4.0, 5.0, and 6.0 (Figure 3A). The decline in the *q*_MO_ observed between pH 4.0 and 6.0 can be attributed to a reduction in the positive charge on the microspheres, weakening the electrostatic attraction. Beyond a pH of 6.6, the electrostatic repulsion between MO and the chitosan–PABC composite microspheres intensified, yet the *q*_MO_ remained approximately at 40 mg/g. This phenomenon is likely due to π–π interactions, hydrophobic interactions or hydrogen bonding between the two materials [33,42].

#### 2.2.2. Effect of Dosage

**Figure 4:**
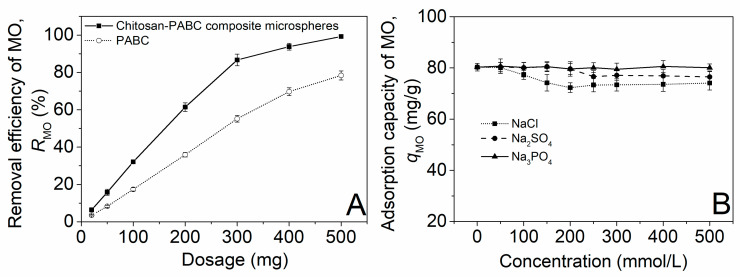
Effects of dosage of PABC and chitosan–PABC composite microspheres on removal efficiency of MO (**A**) and effects of salt type and salt concentration on adsorption capacity of MO on chitosan–PABC composite microspheres (**B**).

In this section, the effect of the dosage of the chitosan–PABC composite microspheres on the removal efficiency of MO (*R*_MO_) was evaluated. This assessment was conducted at an initial pH of 4.0, an initial MO concentration of 50 mg/L and a temperature of 25.0 °C without introducing any salts. The dosage effect of the PABC served as a comparative benchmark. The data depicted in Figure 4A reveal that the *R*_MO_ values achieved with the PABC were significantly inferior compared to those obtained with the chitosan–PABC composite microspheres. This observation underscores the superior adsorption efficacy of the chitosan–PABC synergistic combination over PABC alone in removing MO. This was primarily due to the synergistic effects between the biochar’s porous structure, which provided a large surface area for adsorption, and chitosan’s functional groups, such as amino and hydroxyl groups, that facilitated stronger chemical interactions with the dye molecules, as described in Section 2.1. Furthermore, a marked enhancement in *R*_MO_ was observed, escalating from 6.4 ± 0.9% to 99.2 ± 0.3% as the dosage of microspheres was increased from 20 mg to 500 mg. This tendency was in agreement with the variation in *R*_MO_ with the dosage of wattle bark biochar [42] and was attributed to the increase in adsorption sites with the microsphere dosage.

#### 2.2.3. Effects of Salt Type and Concentration

The presence of salt ions often has a negative effect on dye adsorption [43]. Thus, we herein explored the effects of the salt type and concentration on the adsorption of MO on the chitosan–PABC composite microspheres at an initial pH of 4.0, a microsphere dosage of 100 mg, an initial MO concentration of 50 mg/L and a temperature of 25.0 °C. The results in Figure 4B show that the *q*_MO_ exhibited no significant changes with the increase in the Na_3_PO_4_ concentration, while it slightly reduced as the concentrations of NaCl and Na_2_SO_4_ increased. The above phenomenon resulted from the better abilities of the Cl^−^ and SO_4_^2−^ ions to shield the charges on the microsphere surface than the PO_4_^3−^ ions, thus more readily weakening the electrostatic attraction between the MO and the microsphere [44,45]. Overall, the chitosan–biochar composite microspheres were relatively robust in the presence of different salts and concentrations, maintaining a consistent adsorption capacity for MO.

### 2.3. Adsorption Kinetics, Isotherms and Thermodynamics

#### 2.3.1. Adsorption Kinetics

**Figure 5:**
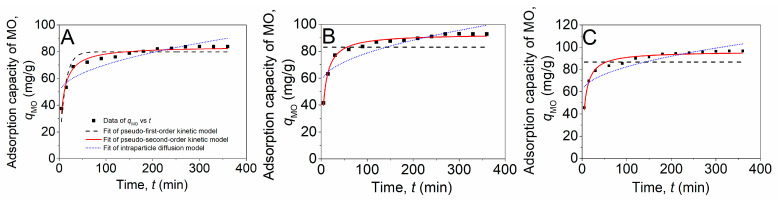
Fitting plots of pseudo-first-order kinetic model, pseudo-second-order kinetic model and intraparticle diffusion model for MO adsorption onto chitosan–PABC composite microspheres at 25 (**A**), 35 (**B**) and 45 °C (**C**).

To further investigate the adsorption dynamics of MO of the chitosan–PABC composite microspheres, we monitored the variations in the adsorption capacity of MO (*q*_MO_) with time. Figure 5 illustrates that the *q*_MO_ experienced a rapid increase followed by a gradual ascent to a plateau as the time interval extended from 5 min to 120 min and subsequently to 360 min across various temperatures. The initial swift rise in the *q*_MO_ could be attributed to the abundance of vacant adsorption sites within the composite microspheres and the high concentration of MO in the solution during the early stage of adsorption [46]. As the availability of these sites and the MO concentration began to diminish, the rate of adsorption progressively decreased [47]. Furthermore, at temperatures of 25.0 (Figure 5A), 35.0 (Figure 5B) and 45.0 °C (Figure 5C), the *q*_MO_ values stabilized at 83.9, 92.9 and 96.7 mg/g, respectively, indicating that an increase in temperature facilitates the adsorption of MO onto the composite microspheres.

To elucidate the kinetics of the adsorption process, the data presented in Figure 5 were analyzed using three conventional adsorption kinetic models: the pseudo-first-order, the pseudo-second-order and the intraparticle diffusion models, with their respective equations detailed in Equations (1)–(3) [48].

(1) $$q_{\mathrm{MO}}=q_{e(\mathrm{MO})}(1-e^{-K_{1}t})$$

(2) $$q_{\mathrm{MO}}=\frac{K_{2}q_{{e(\mathrm{MO})}^{2}t}}{1+K_{2}q_{e(\mathrm{MO})t}}$$

(3) $$q_{\mathrm{MO}}={K_{\mathrm{dif}t}}^{0.5}+\theta$$

where *k*_1_ (min^−1^), *k*_2_ (g/(mg·min)) and *k*_dif_ ((mg/(g·min^−1/2^))) represent the rate constants of the pseudo-first-order kinetic, pseudo-second-order kinetic and intraparticle diffusion models, respectively; *q*_e(MO)_ is the equilibrium absorption capacity of MO; *t* (min) is time; and *θ* (mg/g) is the intraparticle diffusion model’s intercept.

The fitting plots and parameter estimation for the three kinetic models are depicted in Figure 5 and Table 1. According to Table 1, the determination coefficient (*R*^2^) for the pseudo-second-order kinetic model exceeded 0.97 at all temperatures, substantially surpassing those of the pseudo-first-order and intraparticle diffusion models. This indicates that the adsorption behavior of MO of the chitosan–PABC composite microspheres is most accurately described by the pseudo-second-order kinetic model. This result was consistent with that for the adsorption of MO onto iron-loaded sludge and liriodendron leaves biochar [32] and also waste bamboo biochar [49]. In addition, it was observed that the *k*_2_ value increased with temperature, suggesting that elevated temperatures enhanced the adsorption efficiency of MO on the chitosan–PABC composite microspheres.

#### 2.3.2. Adsorption Isotherms

**Figure 6:**
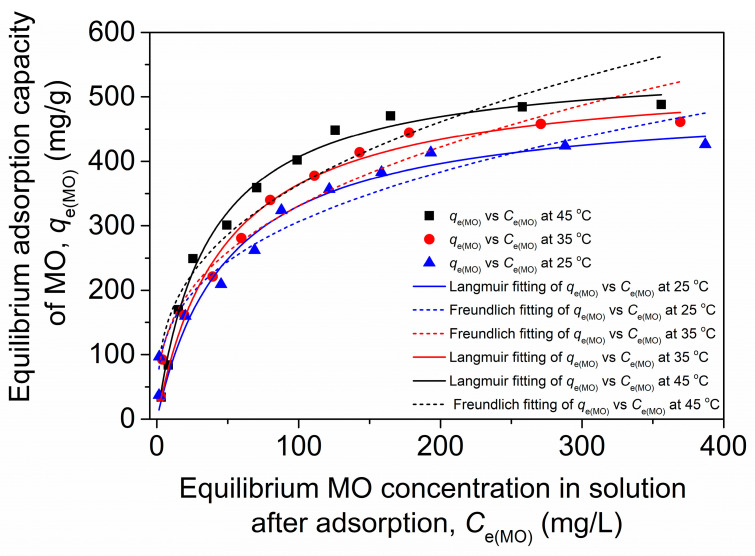
Fitting plots of Langmuir model and Freundlich model adsorption isotherms for MO adsorption onto chitosan–PABC composite microspheres.

In this section, we explored the adsorption isotherms of MO of the chitosan–PABC composite microspheres by examining the variations in the equilibrium absorption capacity of MO (*q*_e(MO)_) with an equilibrated MO concentration in the solution after adsorption (*C*_e(MO)_), as presented in Figure 6. The analysis reveals that *q*_e(MO)_ increased rapidly at first and then more gradually, approaching an asymptote as *C*_e(MO)_ rose at each temperature increment. Notably, the asymptotic value of *q*_e(MO)_ escalated with an increase in temperature. To delve deeper into these observations, the Langmuir and Freundlich isotherm models were employed for data fitting, with their equations denoted as Equations (4) and (5), respectively [34].

(4) $$q_{e(\mathrm{MO})}=\frac{q_{\max(\mathrm{MO})}K_{L}C_{e(\mathrm{MO})}}{1+K_{L}C_{e(\mathrm{MO})}}$$

(5) $$q_{e(\mathrm{MO})}=K_{F}{C_{e(\mathrm{MO})}}^{1/n}$$

where *q*_max(MO)_ (mg/g) is the maximal adsorption capacity of MO on the chitosan–PABC composite microspheres, *K*_L_ (L/mg) is the Langmuir equilibrium constant, and *K*_F_ and 1/n are the Freundlich constants pertaining to the adsorption capacity and the intensity of sorption, respectively.

The fitting plots and calculated parameters are detailed in Figure 6 and Table 2. The Langmuir isotherm model’s determination coefficient (*R*^2^) values approached closer to 1 than those of the Freundlich isotherm model across all the temperatures examined. Consequently, the Langmuir isotherm provided a superior fit for the adsorption behavior of MO of the chitosan–PABC composite microspheres. The maximal MO adsorption capacities (*q*_max(MO)_) determined through the Langmuir model were notably high, registering at 495.0, 537.1 and 554.3 mg/g at 25.0, 35.0 and 45.0 °C, respectively. These capacities exceeded the majority of those documented in the literature, as collated in Table 3. The main reason for this might be that the chitosan–PABC composite microspheres had more functional groups that could be associated with MO through various interactions than the reported absorbents in Table 3. It is suggested that the chitosan–PABC composite microspheres are a highly efficient adsorbent for the removal of MO from dyeing wastewater.

Furthermore, the separation factor, *R*_L_ (dimensionless), was calculated using Equation (6) [51] and served as an indicator of the adsorptive interaction’s nature within the system, as per Langmuir’s theory.

(6) $$R_{L}=\frac{1}{1+K_{L}C_{0}}$$

where *C*_0_ (mg/L) is the initial adsorbate concentration and herein equals 200 mg/L. Generally, the nature of the adsorption isotherm was deemed favorable if the *R*_L_ values range between 0 and 1 [59]. The *R*_L_ values for the composite microspheres were recorded in Table 2 as 0.199, 0.172 and 0.153 at 25.0, 35.0 and 45.0 °C, respectively. These data indicate a favorable adsorption process for MO within the microspheres.

#### 2.3.3. Adsorption Thermodynamics

The results in Section 2.3.1 and Section 2.3.2 demonstrated that elevating the temperature not only accelerated the adsorption rate of MO of the chitosan–PABC composite microspheres but also improved the equilibrium absorption capacity. On this basis, the thermodynamics for this adsorption process were analyzed using the Gibbs free energy change equation and the Van’t Hoff equation defined as Equations (7) and (8), respectively [59].

(7) ΔG=ΔH−TΔS

(8) $$\ln K_{d}=-\frac{\Delta H}{RT}+\frac{\Delta S}{R}$$

where Δ*G* (KJ/mol), Δ*H* (KJ/mol) and Δ*S* (KJ/(mol·K)) represent the changes in the Gibbs free energy, enthalpy and entropy, respectively; *R* is the perfect gas constant, being 8.314 J/(mol·K).

*K*_d_ is the dimensionless distribution coefficient of adsorption defined as Equation (9) [60], and *T* (K) is temperature.

(9) $$K_{d}=K_{L}\times 1000$$

Based on the data of temperature vs. *K*_L_, Δ*G*, Δ*H* and Δ*S* were calculated and are described in Table 4. It is presented that the values of ΔG are negative at all the temperatures. It is indicated that the adsorption of MO onto the chitosan–PABC composite microspheres was spontaneous. With an increase in temperature, the absolute value of ΔG increased (from −7.47 KJ/mol to −8.81 KJ/mol), suggesting that an increase in temperature is favorable for the adsorption process. The positive value of ΔH (12.52 KJ/mol) suggests that the adsorption process is endothermic. This is usually associated with an increase in intermolecular interactions or the formation of chemical bonds during adsorption [47]. Moreover, ΔS is positive (0.067 KJ/(mol·K)), indicating that the disorder of the system increased during the adsorption process. This might be because the MO molecules moved from the solution to the fixed adsorbent surface, and the water molecules in the solution gained more freedom of movement due to the removal of MO molecules, thus leading to an overall increase in the system’s disorder [33]. These data were similar to those for the adsorption of reactive brilliant blue on chitosan–rice husk biochar hydrogel beads [34]. In summary, the adsorption of MO onto the chitosan–PABC composite microspheres was a spontaneous and endothermic process with increased system disorder.

### 2.4. Adsorption Mechanisms

**Figure 7:**
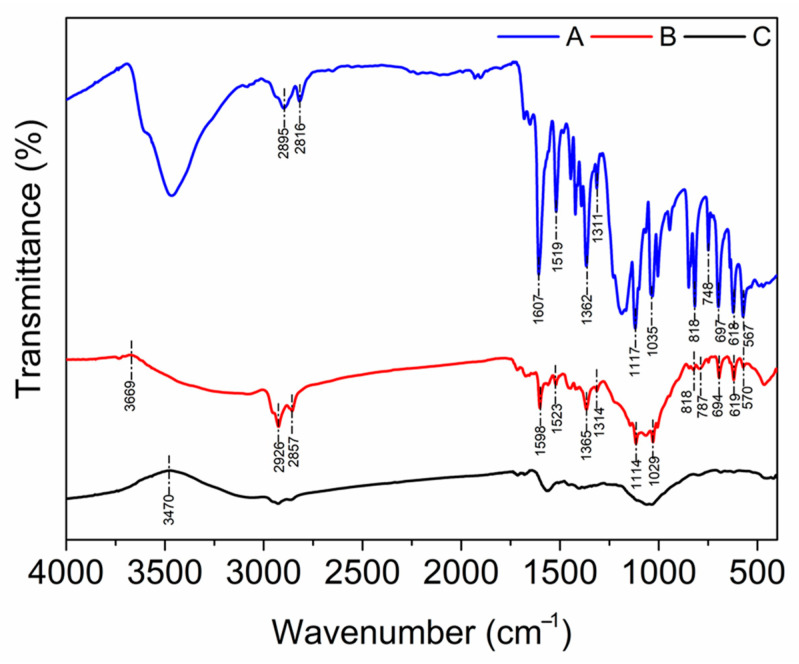
FTIR spectra of MO (A), chitosan–PABC composite microspheres after the adsorption of MO (B) and chitosan–PABC composite microspheres before the adsorption of MO (C).

**Figure 8:**
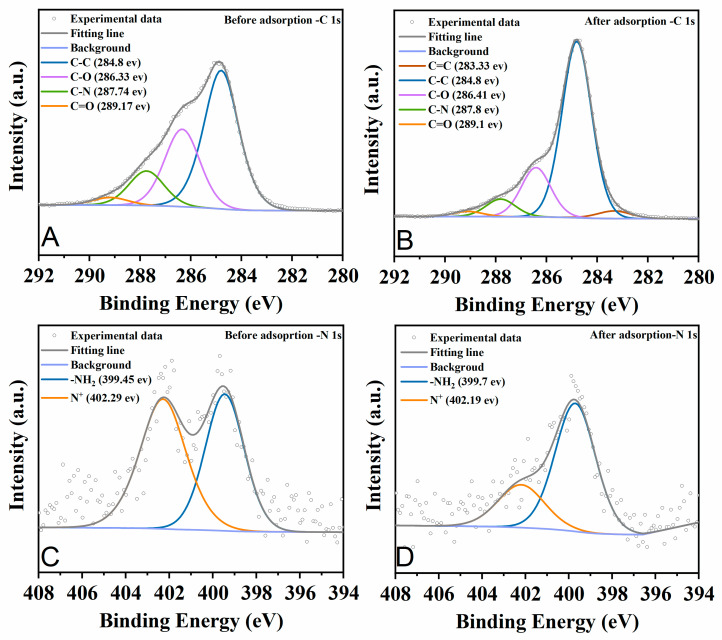
C 1 s XPS spectra of chitosan–PABC composite microspheres before (**A**) and after (**B**) adsorption of MO; N 1 s XPS spectra of chitosan–PABC composite microspheres before (**C**) and after (**D**) adsorption of MO.

To elucidate the efficient adsorption of MO onto the chitosan–PABC composite microspheres, we investigated the adsorption mechanisms through FTIR and XPS analyses. The findings are delineated in Figure 7 and Figure 8. An analysis of Figure 7 reveals a discernible shift of the upward convex peak from 3470 cm^−1^ to 3669 cm^−1^ in the FTIR spectrum post-MO adsorption, accompanied by a diminution in peak area. This shift signifies the disruption of internal hydrogen bonds within the microspheres and the concurrent formation of new hydrogen bonds between MO and the microsphere surface [42]. A comparative study of the spectra before and after MO adsorption unveils an augmentation in the peak areas corresponding to the –CH_3_ and –CH_2_ groups (2926 and 2857 cm^−1^). Moreover, the characteristic stretching vibrations of MO, including C=C (1607 cm^−1^), -SO_3_^−^ (1362 cm^−1^), C-N (1311 cm^−1^), C-O-C (1117 and 1035 cm^−1^), and those pertaining to the aromatic ring (1519, 818, 748, 697, 618, and 567 cm^−1^), emerged after adsorption but were absent in the spectrum before adsorption. These spectral signatures corroborated the successful adsorption of MO onto the chitosan–PABC composite microspheres. In addition, these peaks exhibited varying extents of shifting from those observed in MO’s spectrum alone, implying that the -SO_3_^−^, C-N and C-O-C groups and the aromatic rings participated in the adsorption process through electrostatic attraction, hydrophobic interactions and π–π interactions [61,62].

Figure 8 elucidates the C1s and N1s spectra of the chitosan–PABC composite microspheres before and after MO adsorption. Before adsorption, the C1s spectra (Figure 8A) reveal peaks at 284.8, 286.33, 287.74 and 289.17 eV, corresponding to the C–H, C–O, C–N and C=O bonds, respectively [34,63]. The N1s spectra before adsorption (Figure 8C) display peaks at 399.45 and 402.29 eV, indicative of –NH_2_ and N^+^ bonds [64]. After adsorption, an augmentation in the C=C peak at 283.33 eV is noted in Figure 8B, possibly due to the adsorbed MO or π–π interactions between MO and the microspheres. Concurrently, a reduction in the molar ratio of C–O (from 29.73% to 19.22%), C–N (from 13.26% to 6.84%), C=O (from 2.91 to 2.01) and N^+^ (from 53.47% to 27.92%), alongside an increase in –NH_2_ (from 46.53% to 72.08%), was observed. This suggests the involvement of C–O, C–N, C=O, –NH_2_ and N^+^ bonds in MO adsorption, potentially facilitated by hydrogen bonding and electrostatic attractions between MO and the microspheres. These findings aligned with those presented in Figure 7.

Overall, the adsorption mechanisms were predominantly governed by electrostatic interactions between the ionized -SO_3_^−^ groups of MO at a pH of 4.0 and the -NH_3_^+^ groups present on the composite microspheres. In addition, hydrogen bonding between the –NH_2_ groups on MO and the oxygen-containing functional groups on the composite microspheres, alongside the π–π interactions and hydrophobic interactions between the benzene rings of MO and the composite microspheres, played significant roles in facilitating the adsorption process.

### 2.5. Regeneration of Chitosan–PABC Composite Microspheres

**Figure 9:**
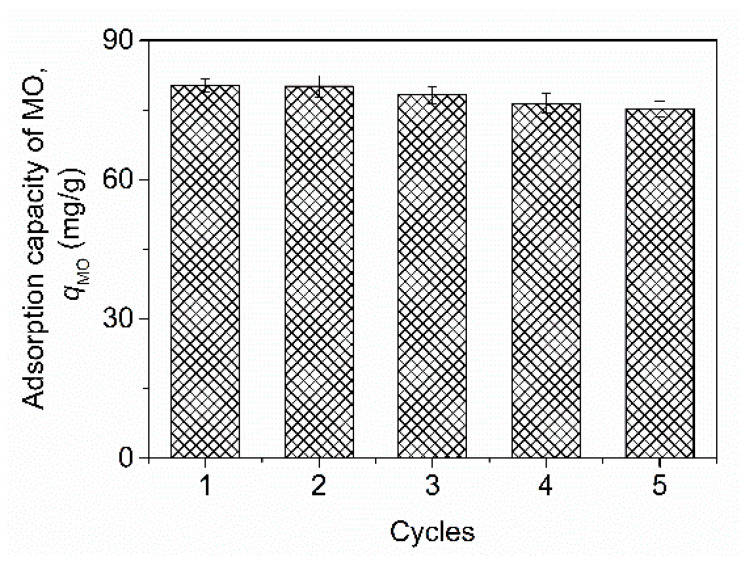
The adsorption capacity of MO on chitosan–PABC composite microspheres in five regeneration cycles.

Considering the imperatives of economic efficiency and environmental stewardship, the regeneration of adsorbents is of significant importance. Consequently, this section delved into the regeneration process of the chitosan–PABC composite microspheres, achieved through the desorption of MO utilizing a 1 mol/L NaOH solution. The microspheres that had adsorbed MO were procured from adsorption experiments conducted at a microsphere dosage of 100 mg, an initial MO concentration of 50 mg/L, an initial pH of 4.0, an adsorption time of 2 h and a temperature of 25 °C, without the addition of salt. Figure 9 graphically depicts the obtained results. Upon undergoing five cycles of reuse, the microspheres demonstrated an average adsorption capacity for MO of 75.2 mg/g, which corresponds to 93.6% of their original mean adsorption capacity. These outcomes underscore the efficacy of the chitosan–PABC composite microspheres as adsorbents for the removal of MO from wastewater, thereby affirming their potential as a dependable and sustainable alternative to the removal of anionic azo dyes from dyeing wastewater.

## 3. Materials and Methods

### 3.1. Materials

Palmer amaranth without roots was harvested from the cultivated land of XiaoZoujia village, Liaocheng, Shandong Province, China, in May 2023. Chitosan with a degree of deacetylation of 80.0–95.0% and a viscosity of 50–800 mPa·s was purchased from Sinopharm Chemical Reagent Co., Ltd., Shanghai, China. Acetic acid, liquid paraffin, glutaraldehyde, acetone, ethanol, MO and other chemical reagents were of analytical grade and obtained from Aladdin Biochemical Technology Co., Ltd., Shanghai, China. Ultrapure water with an electrical resistance of 18.2 MΩ was employed throughout all the experiments.

### 3.2. Preparation of PABC

The PABC was prepared through a slow pyrolysis process according to Ronsse et al. [65] with some modifications. First, the harvested weeds were initially washed, air-dried, and then crushed. Second, the crushed weeds underwent secondary grinding using a high-speed grinder (HCP-100, Yongkang Jinsui Machinery Factory, Yongkang, China) and were passed through a sieve with a mean pore diameter of 2.0 mm. Third, the resulting weed powders were further dried at 80 °C for 4 h in a vacuum drying oven (DZF-6032, Shanghai Yiheng Scientific Instrument Co., Ltd., Shanghai, China). Fourth, the dried weed powders were transferred to a CY-PY1100C-S microwave pyrolysis furnace (Hunan Changyi Microwave Technology Co., Ltd., Changsha, China), and the air in the furnace chamber was purged with nitrogen at a flow rate of 500 mL/min at room temperature (25 °C) for 30 min. Fifth, the powders were then heated up to 500 °C at a heating rate of 10 °C/min and maintained at the final temperature for 70 min. Sixth, the furnace chamber was cooled down to room temperature using the flowing nitrogen and the prepared biochar was collected. After collection, the biochar was further ground and then screened using a sieve with a mean pore diameter of 37.5 μm. Finally, the biochar particles with mean size ≤ 37.5 μm were stored in a glass desiccator at room temperature until further use.

### 3.3. Preparation of Chitosan–PABC Composite Microspheres

The procedural steps for the synthesis of chitosan–PABC composite microspheres are delineated on the basis of Zhao et al. [34]. Initially, 1.0 g of chitosan was introduced into 50 mL of a 2% (*v*/*v*) acetic acid solution. The mixture was then stirred at 200 rpm using an OS-400 Pro stirrer (Hangzhou Xiniu Technology Co., Ltd., Hangzhou, China) until achieving a state of transparency. Following this, 2.0 g of PABC was incorporated into the stirred solution and thoroughly homogenized for 30 min. Subsequently, 1.2 g of Span-80 and 3 mL of glutaraldehyde were added into 80 mL of liquid paraffin. The mixture was mixed at 60 °C within a constant-temperature water bath for 5 min under agitation at 200 rpm. Upon achieving uniformity, its pH was adjusted to a range of 9–10 using 3 mmol/L NaOH solution. Thereafter, 10 mL of the mixed biochar–chitosan solution was extracted via a syringe and dispensed into the aforementioned liquid mixture for reaction, with the stirrer speed set to 200 rpm. The reaction was sustained for 24 h until the supernatant attained clarity and transparency. Finally, upon cessation of the reaction, the supernatant was discarded, and the resulting microspheres were subjected to successive washes with acetone, ethanol, and water, each performed five times. The washed microspheres were dried in a 101-00B electric thermostatic drying oven (Yuyao Sstar Instrument Factory, Yuyao, China) set to 150 °C and then placed in a sealed centrifuge tube at room temperature for use.

### 3.4. Characterizations of PABC and Chitosan–PABC Composite Microspheres

The TESCAN MIRA LMS scanning electron microscope (TESCAN, Brno, Czech Republic) was utilized to examine the morphology and microstructure of PABC and chitosan–PABC composite microspheres. The surface area and pore volume of the composite microspheres were quantified employing the BET method, specifically using the TriStar II 3020 apparatus from Mike, Atlanta, GA, USA. Additionally, FTIR assessments with a wavenumber span of 400 to 4000 cm^−1^ were conducted utilizing the Nicolet iS20 instrument from Thermo Fisher Scientific, Waltham, MA, USA, to delineate the functional groups present on the PABC’s and microspheres’ surfaces, both prior to and subsequent to the adsorption of MO. Before the FTIR measurements, aliquots of the dried samples and potassium bromide powder were mixed in a mortar and subjected to extensive grinding through multiple iterations. Subsequently, the homogenized mixture was compacted into a translucent thin section utilizing a pelleting press. XPS investigations were carried out with the K-Alpha instrument (Thermo Fisher Scientific, USA) to elucidate the valence states of elements on the PABC’s and microspheres’ surfaces before and after MO adsorption, under a background vacuum in the analysis chamber at 2.0 × 10^−7^ mbar and utilizing an Al Kα ray excitation source with an energy of 1361 eV. The point of zero charge (pH_PZC_) under varying pH conditions for CBHBs was determined via the pH drift technique as described by Salimi et al. [66].

### 3.5. Measurement of MO Concentration

The MO concentration was measured by a spectrophotometric method using a U-3900H ultraviolet-visible spectrophotometer (Hitachi, Tokyo, Japan). The standard equation was *A* = 0.2388*C*_MO_-0.0082, *R*^2^ = 0.9998, where *A* is the absorbance at the max wavelength of 463 nm and *C*_MO_ is the concentration of MO ranging from 0 to 5.0 mg/L.

### 3.6. Batch Adsorption Experiments

Adsorption experiments for MO in aqueous solutions were conducted using a batch method. These investigations aimed to assess the impact of various parameters, including the pH range of 2 to 10, the dosages of chitosan–PABC composite microspheres and PABC varying from 20 to 500 mg, the presence of different salts (NaCl, Na_3_PO_4_ and Na_2_SO_4_), and salt concentrations ranging from 0 to 500 mmol/L. To facilitate this, either PABC or chitosan–PABC composite microspheres were introduced into 500 mL of a 50 mg/L MO aqueous solution contained within a 1000 mL Erlenmeyer flask, which was then sealed with plastic wrap. The flask was placed on an SHJ-3 thermostatic magnetic stirrer (manufactured by Changzhou Danrui Experimental Equipment Co., Ltd., Changzhou, China) and agitated at a constant temperature of 25.0 °C and a speed of 200 rpm for 3 h.

Kinetic studies were performed at intervals of 5,15, 30, 60, 90, 120, 150, 180, 210, 240, 270, 300, 330 and 360 min, maintaining an MO concentration of 50 mg/L, a chitosan–PABC composite microsphere dosage of 100 mg, pH 4.0, and temperatures of 25, 35 and 45 °C, without the addition of any salts. Isotherm studies were conducted at MO concentrations of 20, 50, 100, 150, 200, 250, 300, 350, 400, 500 and 600 mg/L, using a chitosan–PABC composite microsphere dosage of 100 mg, at pH 4.0, an adsorption duration of 4 h, and temperatures of 25, 35 and 45 °C, also without the addition of any salts.

Removal efficiency and adsorption capacity of MO (*R*_MO_ and *q*_MO_) were used to evaluate the adsorption process and are defined as Equation (10) and Equation (11), respectively.

(10) $$R_{\mathrm{MO}}=(1-\frac{C_{r(\mathrm{MO})}}{C_{o(\mathrm{MO})}})\times 100\%$$

(11) $$q_{\mathrm{MO}}=\frac{(C_{o(\mathrm{MO})}-C_{r(\mathrm{MO})})V}{m}$$

where *C*_o(MO)_ and *C*_r(MO)_ (mg/L) are concentrations of MO before and after adsorption, *V* is the volume of MO solution, 500 mL and *m* (g) is the mass of chitosan–PABC composite microspheres.

### 3.7. Desorption and Regeneration Studies

Upon completion of the adsorption procedure, the chitosan–PABC composite microspheres were isolated from the residual solution of MO and thereafter subjected to rinsing with ultrapure water. For the desorption of the adsorbed MO from the microspheres, a solution of 1.0 mol/L NaOH served as the eluent, facilitating the regeneration of the microspheres. These regenerated microspheres were then employed in successive adsorption trials for subsequent cycles.

### 3.8. Statistical Analysis

Each experiment was executed with at least three replicates. The analysis of data was performed utilizing the Excel application of Microsoft Office Professional Plus 2013 (Microsoft, Redmond, WA, USA), adopting a significance threshold of *p* ≤ 0.05. Standard deviations were computed and presented alongside each mean value.

## 4. Conclusions

Leveraging the undesirable proliferation of Palmer amaranth, this study transformed the weed into a beneficial adsorbent, i.e., chitosan–PABC composite microspheres for the removal of MO from dyeing wastewater. The successful preparation of the composite microspheres was demonstrated by SEM, FTIR and BET analyses. The process of MO adsorption onto the microspheres was optimized at pH 4.0, demonstrating the significant influence of the pH, adsorbent dosage, and presence of salts on the efficiency of MO removal. The kinetic and isotherm models indicate that the adsorption follows pseudo-second-order kinetics and fits the Langmuir isotherm, suggesting monolayer adsorption with a maximal capacity of 495.0–554.3 mg/g. The thermodynamic analysis confirms that the adsorption process is spontaneous and endothermic. The adsorption mechanism was predominantly attributed to electrostatic attraction, hydrogen bonding, π–π interactions and hydrophobic interactions between the composite microspheres and MO molecules. Furthermore, the composite microspheres showed an excellent regeneration capability, maintaining significant adsorption efficiency across multiple cycles. When they are not renewable, they could be burned to produce thermal energy and the ashes used as a soil amendment. This study not only addressed environmental concerns associated with MO pollution but also proposed a sustainable strategy for managing the proliferation of Palmer amaranth, highlighting the potential of biochar-based materials in water purification technologies.

## Figures and Tables

**Table 1:** Kinetic parameters for pseudo-first-order kinetic model, pseudo-second-order kinetic model and intraparticle diffusion model calculated from the fitting results in Figure 5.

Temperature (°C)		25	35	45
Pseudo-first-order kinetic model	*q*_e(MO)_ (mg/g)	79.9	83.0	86.7
	*K*_1_ (min^−1^)	0.0851	16.1	17.6
	*R* ^2^	0.8618	0.08333	0.08333
Pseudo-second-order kinetic model	*q*_e(MO)_ (mg/g)	84.0	92.9	96.3
	*K*_2_ (g/(mg·min))	1.61 × 10^−7^	1.66 × 10^−7^	1.73 × 10^−7^
	*R* ^2^	0.97149	0.98364	0.97133
Intraparticle diffusion model	*K*_dif_ (mg/(g·min^−1/2^))	2.20	2.30	2.31
	Θ (mg/g)	48.3	55.6	59.3
	*R* ^2^	0.76691	0.73244	0.75701

**Table 2:** Isotherm parameters for Langmuir and Freundlich models calculated from the fitting results in Figure 6.

Temperature (°C)	Langmuir				Freundlich		
	*K*_L_ (L/mg)	*q*_max(MO)_ (mg/g)	*R*_L_	*R* ^2^	*K*_F_ (mg^(1−n)^ L^n^/g)	1/n	*R* ^2^
25	0.0201	495.0	0.199	0.94578	68.8	0.324	0.94482
35	0.0241	537.1	0.172	0.97935	65.6	0.351	0.93311
45	0.0276	554.3	0.153	0.99144	74.6	0.344	0.89581

**Table 3:** The maximal adsorption capacities of MO (*q*_max(MO)_) of biochar-derived adsorbents reported in previous literature and this work.

Adsorbent	*q*_max(MO)_ (mg/g)	Reference
Chicken manure biochar	39.47	[16]
Chitosan-modified rice husk biochar	38.75	[17]
Sheep manure biochar	42.513~45.563	[18]
Biochar derived from date palm petioles	461	[21]
Waste bamboo-derived biochar	344.8	[49]
Zinc-loaded chitosan biochar	120.92	[50]
EDTA and chitosan bi-functionalized magnetic bamboo biochar	305.4	[51]
Pine cone biochar activated with KOH	109.5	[52]
Biochar of the secondary medicinal residue of snow lotus	480~640	[53]
Sulfur-modified nZVI supported on biochar composite	622.58	[54]
Activated biochar derived from pomelo peel wastes	163.105	[55]
Multi-porous biochar from lotus biomass	320	[56]
Fe_3_O_4_-modified magnetic mesoporous biochar	392.06	[57]
Mg/Al-LDHs–biochar hybrid materials	75.98	[58]
Chitosan–PABC composite microspheres	495.0~554.3	This work

**Table 4:** The calculated thermodynamic parameters for MO adsorption onto chitosan–PABC composite microspheres.

T (K)	Δ*G* (KJ/mol)	Δ*H* (KJ/mol)	Δ*S* (KJ/(mol·K))
298.15	−7.47	12.52	0.067
308.15	−8.14		
318.15	−8.81		

## Data Availability

The data are contained within the article.

## Supplementary Materials

The following supporting information can be downloaded at https://www.mdpi.com/article/10.3390/molecules29081836/s1. Figure S1. Isotherm linear plot of chitosan–Palmer amaranth biochar composite microspheres (A); BJH desorption dV/dw pore volume plot of chitosan–Palmer amaranth biochar composite microspheres (B).

## References

[1] Priyanka U., Lens P.N. (2022). Light driven *Aspergillus niger*-ZnS nanobiohybrids for degradation of methyl orange. Chemosphere.

[2] Iwuozor K.O., Ighalo J.O., Emenike E.C., Ogunfowora L.A., Igwegbe C.A. (2021). Adsorption of methyl orange: A review on adsorbent performance. Curr. Res. Green Sustain. Chem..

[3] Ismail M., Akhtar K., Khan M.I., Kamal T., Khan M.A., Asiri A.M., Seo J., Khan S.B. (2019). Pollution, toxicity and carcinogenicity of organic dyes and their catalytic bio-remediation. Curr. Pharm. Des..

[4] Jankowska A., Ejsmont A., Galarda A., Goscianska J. (2022). The outcome of human exposure to environmental contaminants. Importance of water and air purification processes. Sustainable Materials for Sensing and Remediation of Noxious Pollutants.

[5] Saxena R., Saxena M., Lochab A. (2020). Recent progress in nanomaterials for adsorptive removal of organic contaminants from wastewater. ChemistrySelect.

[6] Uddin M.J., Ampiaw R.E., Lee W. (2021). Adsorptive removal of dyes from wastewater using a metal-organic framework: A review. Chemosphere.

[7] Kausar A., Zohra S.T., Ijaz S., Iqbal M., Iqbal J., Bibi I., Nouren S., Messaoudi N.E., Nazir A. (2023). Cellulose-based materials and their adsorptive removal efficiency for dyes: A review. Int. J. Biol. Macromol..

[8] Yudaev P., Butorova I., Stepanov G., Chistyakov E. (2022). Extraction of palladium (ii) with a magnetic sorbent based on polyvinyl alcohol gel, metallic iron, and an environmentally friendly polydentate phosphazene-containing extractant. Gels.

[9] Yudaev P., Semenova A., Chistyakov E. (2024). Gel based on modified chitosan for oil spill cleanup. J. Appl. Polym. Sci..

[10] Sayed N.S., Ahmed A.S., Abdallah M.H., Gouda G.A. (2024). ZnO@ activated carbon derived from wood sawdust as adsorbent for removal of methyl red and methyl orange from aqueous solutions. Sci. Rep..

[11] Darwish A.A.A., Rashad M., AL-Aoh H.A. (2019). Methyl orange adsorption comparison on nanoparticles: Isotherm, kinetics, and thermodynamic studies. Dye. Pigment..

[12] Birniwa A.H., Ali U., Jahun B.M., Al-dhawi B.N.S., Jagaba A.H. (2024). Cobalt oxide doped polyaniline composites for methyl orange adsorption: Optimization through response surface methodology. Case Stud. Chem. Environ. Eng..

[13] Wang C., Feng X., Shang S., Liu H., Song Z., Zhang H. (2023). Adsorption of methyl orange from aqueous solution with lignin-modified metal-organic frameworks: Selective adsorption and high adsorption capacity. Bioresour. Technol..

[14] Sutar S., Patil P., Jadhav J. (2022). Recent advances in biochar technology for textile dyes wastewater remediation: A review. Environ. Res..

[15] Cheng N., Wang B., Wu P., Lee X., Xing Y., Chen M., Gao B. (2021). Adsorption of emerging contaminants from water and wastewater by modified biochar: A review. Environ. Pollut..

[16] Yu J., Zhang X., Wang D., Li P. (2018). Adsorption of methyl orange dye onto biochar adsorbent prepared from chicken manure. Water Sci. Technol..

[17] Loc N.X., Tuyen PT T., Mai L.C., Phuong D.T.M. (2022). Chitosan-modified biochar and unmodified biochar for methyl orange: Adsorption characteristics and mechanism exploration. Toxics.

[18] Lu Y., Chen J., Bai Y., Gao J., Peng M. (2019). Adsorption properties of methyl orange in water by sheep manure biochar. Pol. J. Environ. Stud..

[19] Park H., Kim J., Lee Y.G., Chon K. (2021). Enhanced adsorptive removal of dyes using Mandarin peel biochars via chemical activation with NH_4_Cl and ZnCl_2_. Water.

[20] Ighalo J.O., Zhou Y., Zhou Y., Igwegbe C.A., Anastopoulos I., Raji M.A., Iwuozor K.O. (2022). A review of pine-based adsorbents for the adsorption of dyes. Biomass-Deriv. Mater. Environ. Appl..

[21] Aichour A., Zaghouane-Boudiaf H., Khodja H.D. (2022). Highly removal of anionic dye from aqueous medium using a promising biochar derived from date palm petioles: Characterization, adsorption properties and reuse studies. Arab. J. Chem..

[22] Yan X., Sun Y., Ma C., Kong X., Zhang Y., Tao W. (2021). Adsorption of anionic and cationic dyes on different biochars. Russ. J. Phys. Chem. A.

[23] Wang Y., Liu R. (2017). Comparison of characteristics of twenty-one types of biochar and their ability to remove multi-heavy metals and methylene blue in solution. Fuel Process. Technol..

[24] Pariyar P., Kumari K., Jain M.K., Jadhao P.S. (2020). Evaluation of change in biochar properties derived from different feedstock and pyrolysis temperature for environmental and agricultural application. Sci. Total Environ..

[25] Roberts J., Florentine S. (2022). A review of the biology, distribution patterns and management of the invasive species *Amaranthus palmeri* S. Watson (*Palmer amaranth*): Current and future management challenges. Weed Res..

[26] Jiao X., Long M., Li J., Yang Q., Liu Z. (2023). Reconstructing the invasive history and potential distribution prediction of *Amaranthus palmeri* in China. Agronomy.

[27] Danesh P., Niaparast P., Ghorbannezhad P., Ali I. (2023). Biochar production: Recent developments, applications, and challenges. Fuel.

[28] Zhou Y., Qin S., Verma S., Sar T., Sarsaiya S., Ravindran B., Liu T., Sindhu R., Patel A.K., Binod P. (2021). Production and beneficial impact of biochar for environmental application: A comprehensive review. Bioresour. Technol..

[29] Wang Q., Lai Z., Mu J., Chu D., Zang X. (2020). Converting industrial waste cork to biochar as Cu (II) adsorbent via slow pyrolysis. Waste Manag..

[30] Patra B.R., Mukherjee A., Nanda S., Dalai A.K. (2021). Biochar production, activation and adsorptive applications: A review. Environ. Chem. Lett..

[31] Wang J., Wang S. (2019). Preparation, modification and environmental application of biochar: A review. J. Clean. Prod..

[32] Cheng H., Liu Y., Li X. (2021). Adsorption performance and mechanism of iron-loaded biochar to methyl orange in the presence of Cr^6+^ from dye wastewater. J. Hazard. Mater..

[33] Gao N., Du W., Zhang M., Ling G., Zhang P. (2022). Chitosan-modified biochar: Preparation, modifications, mechanisms and applications. Int. J. Biol. Macromol..

[34] Zhao Y., Song Y., Li R., Lu F., Yang Y., Huang Q., Deng D., Wu M., Li Y. (2023). Enhanced reactive brilliant blue removal using chitosan–biochar hydrogel beads. Molecules.

[35] Chia C.H., Gong B., Joseph S.D., Marjo C.E., Munroe P., Rich A.M. (2012). Imaging of mineral-enriched biochar by FTIR, Raman and SEM–EDX. Vib. Spectrosc..

[36] Nair R.R., Mondal M.M., Weichgrebe D. (2020). Biochar from co-pyrolysis of urban organic wastes—Investigation of carbon sink potential using ATR-FTIR and TGA. Biomass Convers. Biorefin..

[37] Lago B.C., Silva C.A., Melo LC A., de Morais E.G. (2021). Predicting biochar cation exchange capacity using Fourier transform infrared spectroscopy combined with partial least square regression. Sci. Total Environ..

[38] Duarte M.L., Ferreira M.C., Marvao M.R., Rocha J. (2002). An optimised method to determine the degree of acetylation of chitin and chitosan by FTIR spectroscopy. Int. J. Biol. Macromol..

[39] Zhang L., Tang S., He F., Liu Y., Mao W., Guan Y. (2019). Highly efficient and selective capture of heavy metals by poly (acrylic acid) grafted chitosan and biochar composite for wastewater treatment. Chem. Eng. J..

[40] Perera H.M., Rajapaksha A.U., Liyanage S., Ekanayake A., Selvasembian R., Daverey A., Vithanage M. (2023). Enhanced adsorptive removal of hexavalent chromium in aqueous media using chitosan-modified biochar: Synthesis, sorption mechanism, and reusability. Environ. Res..

[41] Dakiky M., Khamis M., Manasra A., Takrouri K. (2002). Effect of surfactants on the thermodynamic properties of methyl orange dye in buffered solutions. Color. Technol..

[42] Nguyen X.C., Nguyen T.T.H., Nguyen T.H.C., Van Le Q., Vo T.Y.B., Tran T.C.P., La D.D., Kumar G., VNguyen K., Chang S.W. (2021). Sustainable carbonaceous biochar adsorbents derived from agro-wastes and invasive plants for cation dye adsorption from water. Chemosphere.

[43] Velusamy S., Roy A., Sundaram S., Kumar Mallick T. (2021). A review on heavy metal ions and containing dyes removal through graphene oxide-based adsorption strategies for textile wastewater treatment. Chem. Rec..

[44] Ip A.W.M., Barford J.P., McKay G. (2009). Reactive Black dye adsorption/desorption onto different adsorbents: Effect of salt, surface chemistry, pore size and surface area. J. Colloid Interface Sci..

[45] Kamiyama Y., Israelachvili J. (1992). Effect of pH and salt on the adsorption and interactions of an amphoteric polyelectrolyte. Macromolecules.

[46] Tahiruddin N.S.M., Aziz R.A., Ali R., Taib N.I. (2023). Potential of using jackfruit peel (*Artocarpus heterophyllus*) as green solution for removal of copper (II) and zinc (II) from aqueous solution: Adsorption kinetics, isotherm and thermodynamic studies. J. Environ. Chem. Eng..

[47] Zhu H., Chen S., Duan H., He J., Luo Y. (2023). Removal of anionic and cationic dyes using porous chitosan/carboxymethyl cellulose-PEG hydrogels: Optimization, adsorption kinetics, isotherm and thermodynamics studies. Int. J. Biol. Macromol..

[48] Debnath S., Das R. (2023). Strong adsorption of CV dye by Ni ferrite nanoparticles for waste water purification: Fits well the pseudo second order kinetic and Freundlich isotherm model. Ceram. Int..

[49] Wang S.S., Yan Y.M., Hsu C.H., Lin H.P. (2023). Waste bamboo-derived biochar and multiporous carbon as adsorbents for methyl orange removal. J. Chin. Chem. Soc..

[50] Li X.F., Li R.X., Huang M.M., Feng X.Q. (2023). Superior adsorption performance of zinc-loaded chitosan biochar for methyl orange dye. Russ. J. Phys. Chem. A.

[51] Zhang H., Li R., Zhang Z. (2022). A versatile EDTA and chitosan bi-functionalized magnetic bamboo biochar for simultaneous removal of methyl orange and heavy metals from complex wastewater. Environ. Pollut..

[52] Kaya N., Uzun Z.Y. (2021). Investigation of effectiveness of pine cone biochar activated with KOH for methyl orange adsorption and CO_2_ capture. Biomass Convers. Biorefinery.

[53] Zhang S., Yao Y., Li J., Wang L., Wang X., Tian S. (2023). Multi-factorial investigation of the effect of biochar of the secondary medicinal residue of snow lotus on the adsorption of two azo dyes, methyl red and methyl orange. Microsc. Res. Tech..

[54] Yang L., Gao J., Liu Y., Zhang Z., Zou M., Liao Q., Shang J. (2018). Removal of methyl orange from water using sulfur-modified nZVI supported on biochar composite. Water Air Soil Pollut..

[55] Zhang B., Wu Y., Cha L. (2020). Removal of methyl orange dye using activated biochar derived from pomelo peel wastes: Performance, isotherm, and kinetic studies. J. Dispers. Sci. Technol..

[56] Hou Y., Liang Y., Hu H., Tao Y., Zhou J., Cai J. (2021). Facile preparation of multi-porous biochar from lotus biomass for methyl orange removal: Kinetics, isotherms, and regeneration studies. Bioresour. Technol..

[57] Wang J., Chen W., Zhang M., Zhou R., Li J., Zhao W., Wang L. (2021). Optimize the preparation of Fe_3_O_4_-modified magnetic mesoporous biochar and its removal of methyl orange in wastewater. Environ. Monit. Assess..

[58] Li H., Wu G., Liu Q., Wang J., Xia S., Xue Y., Han J., Duanmuc C., Zhu Y. (2022). A comprehensive insight into removal of dye and antibiotic based on Mg/Al-LDHs-biochar hybrid materials. Mater. Technol..

[59] Karagöz S., Tay T., Ucar S., Erdem M. (2008). Activated carbons from waste biomass by sulfuric acid activation and their use on methylene blue adsorption. Bioresour. Technol..

[60] Lima E.C., Hosseini-Bandegharaei A., Moreno-Piraján J.C., Anastopoulos I. (2019). A critical review of the estimation of the thermodynamic parameters on adsorption equilibria. Wrong use of equilibrium constant in the Van’t Hoof equation for calculation of thermodynamic parameters of adsorption. J. Mol. Liq..

[61] Guo S., Zou Z., Chen Y., Long X., Liu M., Li X., Tan J., Chen R. (2023). Synergistic effect of hydrogen bonding and π-π interaction for enhanced adsorption of rhodamine B from water using corn straw biochar. Environ. Pollut..

[62] Ahmed M.B., Zhou J.L., Ngo H.H., Johir M.A.H., Sun L., Asadullah M., Belhaj D. (2018). Sorption of hydrophobic organic contaminants on functionalized biochar: Protagonist role of π-π electron-donor-acceptor interactions and hydrogen bonds. J. Hazard. Mater..

[63] Xiao F., Cheng J., Cao W., Yang C., Chen J., Luo Z. (2019). Removal of heavy metals from aqueous solution using chitosan-combined magnetic biochars. J. Colloid Interface Sci..

[64] Su X., Wang X., Ge Z., Bao Z., Lin L., Chen Y., Dai W., Sun Y., Yuan H., Yang W. (2024). Koh-activated biochar and chitosan composites for efficient adsorption of industrial dye pollutants. Chem. Eng. J..

[65] Ronsse F., Van Hecke S., Dickinson D., Prins W. (2013). Production and characterization of slow pyrolysis biochar: Influence of feedstock type and pyrolysis conditions. GCB Bioenergy.

[66] Salimi H., Fattah-Alhosseini A., Karbasi M., Nikoomanzari E. (2023). Development of WO_3_-incorporated porous ceramic coating: A key role of WO_3_ nanoparticle concentration on methylene blue photodegradation upon visible light illumination. Ceram. Int..

