# Supplementary material for: Enhanced Adsorption of Methyl Orange from Aqueous Phase Using Chitosan–Palmer Amaranth Biochar Composite Microspheres
Source: Molecules. 2024 Apr 18;29(8):1836. doi: 10.3390/molecules29081836 (PMC11054346; doi:10.3390/molecules29081836)
Supplement: Supplementary file 1 [file molecules-29-01836-s001.zip › molecules-2963960-supplementary.pdf]

Supplementary Materials

Enhanced Adsorption of Methyl Orange from Aqueous Phase Using Chitosan-Palmer Amaranth Biochar Composite Microspheres

School of Biological Science, Jining Medical University, No. 669 Xueyuan Road, Donggang District, Rizhao 276826, China; chenguiling@mail.jnmc.edu.cn (G.C.); yytsdml@163.com (Y.Y.); 13589619982@163.com (X.Z.); 13310637723@163.com (A.Q.); pan2071078420@163.com (X.P.)

***** Correspondence: liufei092531@mail.jnmc.edu.cn (F.L.); ruili061289@163.com or ruili061289@mail.jnmc.edu.cn (R.L.)

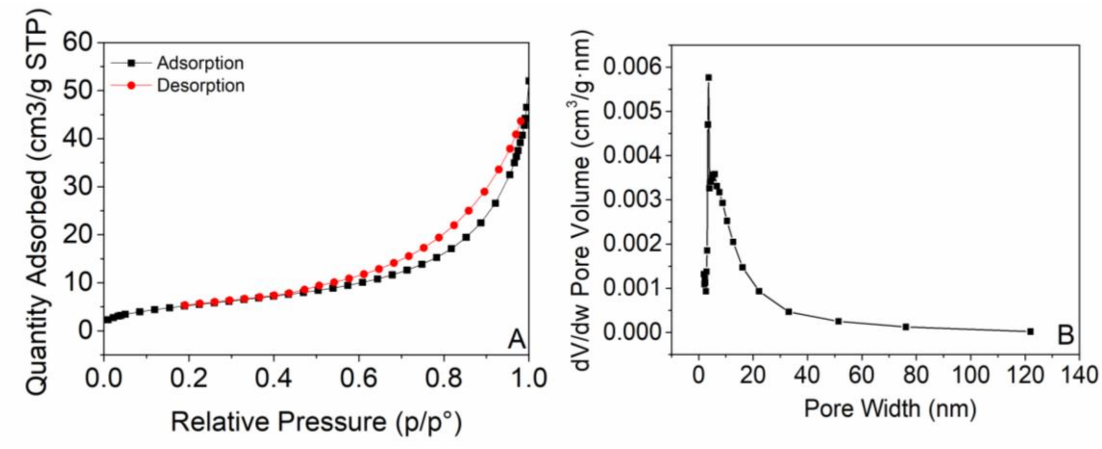

Figure S1. Isotherm linear plot of chitosan- Palmer amaranth biochar composite microspheres (A); BJH desorption dV/dw pore volume plot of chitosan- Palmer amaranth biochar composite microspheres (B).
